# Impact of the Coming‐of‐Age Day and ceremony on the risk of SARS‐CoV‐2 transmission in Japan: A natural‐experimental study based on national surveillance data

**DOI:** 10.1111/irv.13027

**Published:** 2022-07-27

**Authors:** Yura K. Ko, Ryo Kinoshita, Masato Yamauchi, Kanako Otani, Taro Kamigaki, Kazuki Kasuya, Daisuke Yoneoka, Yuzo Arima, Yusuke Kobayashi, Takeshi Arashiro, Miyako Otsuka, Reiko Shimbashi, Motoi Suzuki

**Affiliations:** ^1^ Center for Surveillance, Immunization, and Epidemiologic Research National Institute of Infectious Diseases Tokyo Japan; ^2^ Department of Virology Tohoku University Graduate School of Medicine Sendai Japan; ^3^ Department of Political Science Waseda University Graduate School of Political Science Tokyo Japan

**Keywords:** Coming‐of‐Age Day, COVID‐19, non‐pharmaceutical intervention, SARS‐CoV‐2, social gatherings

## Abstract

**Background:**

Quantifying the impact on COVID‐19 transmission from a single event has been difficult due to the virus transmission dynamics, such as lag from exposure to reported infection, non‐linearity arising from the person‐to‐person transmission, and the modifying effects of non‐pharmaceutical interventions over time. To address these issues, we aimed to estimate the COVID‐19 transmission risk of social events focusing on the Japanese Coming‐of‐Age Day and Coming‐of‐Age ceremony in which “new adults” practice risky behavior on that particular day.

**Methods:**

Using national surveillance data in Japan in 2021 and 2022, we conducted difference‐in‐differences regression against COVID‐19 incidences by setting “new adults” cases as the treatment group and the cases 1 year younger or older than these “new adults” as the control group. In addition, we employed a triple differences approach to estimate the risk of holding the Coming‐Age ceremony by using a binary variable regarding the presence or absence of the ceremony in each municipality.

**Results:**

We estimated the relative risks (RRs) of the Coming‐of‐Age Day as 1.27 (95% confidence interval [CI] 1.02–1.57) in 2021 and 3.22 (95% CI 2.68–3.86) in 2022. The RR of the Coming‐of‐Age ceremony was also large, estimated as 2.83 (1.81–4.43) in 2022.

**Conclusions:**

When planning large social events, it is important to be aware of the unique risks associated with these gatherings, along with effective public health messages to best communicate these risks.

## 1 INTRODUCTION

As of March 31, 2022, over 6,400,000 confirmed cases and 27,000 associated deaths have been reported in Japan due to coronavirus disease (COVID‐19).^1^ As a non‐pharmaceutical intervention (NPI), many countries have implemented public event bans and group size restrictions for social gatherings. NPIs are reportedly associated with a reduced number of COVID‐19 cases and deaths.^2^, ^3^, ^4^ However, quantifying the impact from a single intervention or event is difficult, and there is a lack of relevant evidence. One reason for this is the challenge in setting controls as counterfactuals due to COVID‐19 transmission dynamics (lag from exposure to reported infection, non‐linearity arising from person‐to‐person transmission, and the effects of NPIs having different effects over time).^5^

To address these issues and estimate the risk of infection following social events, we focused on the Japanese Coming‐of‐Age Day (“Seijin‐No‐Hi”), a national holiday celebrated on the second Monday of January. Around this day, the Coming‐of‐Age ceremony (“Seijin‐Shiki”) is conducted for the age cohort who have turned or will turn 20 years old between April 2 of the previous year and April 1 of the current year (new adults). Many “new adults” celebrate with their friends at an izakaya (Japanese‐style pub) after attending the ceremony.^6^, ^7^ This unique setting provides a natural environment to quantify the impact of the social gathering on the transmission risk among this population.

In this study, we aimed to estimate the impact of the Coming‐of‐Age Day on the risk of COVID‐19 transmission in 2021 and 2022 by a difference‐in‐differences (DID) analysis using the number of infections among the new adults (20‐year‐old age cohort) before and after the Coming‐of‐Age Day; the number of infections among persons 1 year younger or older than the new adults (19‐ and 21‐year‐old cohort, respectively), whose attributes are similar to those of the new adults, served as the control group. Some municipalities conducted the Coming‐of‐Age ceremony at a time other than the second Monday of January or postponed the ceremony due to the COVID‐19 pandemic. Therefore, we used a triple‐difference approach^8^ to quantify the impact of conducting the ceremony on the risk of SARS‐CoV‐2 transmission.

## 2 METHODS

### 2.1 Data sources

In Japan, all COVID‐19 cases are registered in the national‐level Health Center Real‐time Information‐sharing System on COVID‐19 (HER‐SYS).^9^ We obtained the daily number of newly reported cases by age (date of birth) and symptom onset date in each municipality using HER‐SYS. Since our target population included individuals celebrating the Coming‐of‐Age Day, we stratified age cohorts by the date of birth between April 2 and April 1 (grade cohort in the Japanese education system, where the school year starts in April). We also collected statistics such as population^10^ and population density^11^ in each municipality as covariates and examined the respective website of all 1916 municipalities to confirm whether the Coming‐of‐Age ceremony was conducted in 2022. We created a binary variable for the occurrence of the ceremony between January 8 and 10. The status of the ceremonies in 2021 could not be obtained.

### 2.2 DID for quantifying the impact of the coming‐of‐age day

We used DID to quantify the impact of the Coming‐of‐Age Day on the reported number of cases among new adults (20‐year‐old age cohort) who were set as the treatment group. Individuals 1 year younger or older than the new adults (19‐ and 21‐year‐old age cohort, respectively) were set as the control group. Over time, secondary transmission from the treatment to the control group was expected. Therefore, we set the outcome as the cumulative number of cases over 4 days rather than the daily number of new cases to include only cases exposed to the Coming‐of‐Age Day as the incubation period (duration between exposure and symptom onset) for COVID‐19 is approximately 3–5 days.^12^, ^13^, ^14^ The quasi‐Poisson regression model with several covariates was used to estimate the impact of the Coming‐of‐Age Day using the following equations:

$$\begin{array}{l} Y_{a,t,i}∼\text{qPoi}\left(\mu_{a,t,i},\phi \mu_{a,t,i}\right),\mu_{a,t,i}=\exp\{\beta_{0}+\beta_{1}{\mathrm{Age}}_{a,t,i}\\ \;+\;\beta_{2}{\text{Time}}_{a,t,i}+\beta_{3}{\mathrm{Age}}_{a,t,i}\times {\text{Time}}_{a,t,i}+\beta_{4}{\text{Intervention}}_{a,t,i}\\ \;+\;\beta_{5}{\text{PopDen}}_{a,t,i}+\beta_{6}{\text{Trend}}_{a,t,i}+\log\left({\text{Pop}}_{a,t,i}\right)\}, \end{array}$$

where $\text{qPoi}\left(\right)$ is the quasi‐Poisson distribution with an over‐dispersion parameter ϕ, $\;Y_{a,t,i}$ is the outcome for the cumulative number of cases in the age cohort a, time t, and municipality i. $\mathrm{Ag}e_{a,t,i}$ is a binary variable indicating whether the observation belongs to the treatment or control group (1: treatment group; 0: control group) and $\mathrm{Tim}e_{a,t,i}$ is a binary variable indicating whether the observation belongs to the period before or after the Coming‐of‐Age Day (0: before; 1: after). Our measure of interest, the impact of the Coming‐of‐Age Day, can be estimated with the coefficient $\beta_{3}$. The covariates include the binary variable on the state of emergency or pre‐emergency measures ( Intervention), a categorical variable indicating an epidemic trend as defined below ( Trend), a continuous variable for population density (persons per square kilometer) ( PopDen), and population ( Pop). We defined the Trend term as four groups: “No epidemic,” fewer than two cases reported in the epidemiological week before the Coming‐of‐Age Day; “Increasing,” the ratio of the number of cases in the epidemiological week before the Coming‐of‐Age Day to the previous week was at least 1.05 times greater; “Decreasing,” the ratio was less than 0.95; and “No change,” the ratio was between 0.95 and 1.05.

We also conducted the same analysis dichotomizing municipalities on “Seijin‐Shiki” status (conducting or not conducting the ceremony).

### 2.3 Triple‐difference analysis for quantifying the impact of the Coming‐of‐Age ceremony

We further estimated the detailed impact of the Coming‐of‐Age ceremony by conducting the triple‐difference analysis, which incorporates the binary variable of whether or not the ceremony was conducted. The quasi‐Poisson regression model with the following equation was used:

$$Y_{a,t,i}∼\text{qPoi}\left(\mu_{a,t,i},\phi \mu_{a,t,i}\right),$$

$$\mu_{a,t,i}=\begin{array}{l} \exp\{\gamma_{0}+\gamma_{1}{\mathrm{Age}}_{a,t,i}+\gamma_{2}{\text{Time}}_{a,t,i}+\gamma_{3}S_{a,t,i}+\gamma_{4}\;{\mathrm{Age}}_{a,t,i}\\ \times {\text{Time}}_{a,t,i}+\gamma_{5}\mathrm{Tim}e_{a,t,i}\times S_{a,t,i}+\gamma_{6}{\mathrm{Age}}_{a,t,i}\times S_{a,t,i}\\ +\;\gamma_{7}{\mathrm{Age}}_{a,t,i}\times {\text{Time}}_{a,t,i}\times S_{a,t,i}+\gamma_{8}{\text{Intervention}}_{a,t,i}+\gamma_{9}\\ \times {\text{PopDen}}_{a,t,i}+\gamma_{10}\times {\text{Trend}}_{a,t,i}+\log\left({\text{Pop}}_{a,t,i}\right)\}, \end{array}$$

where $S_{a,t,i}$ is a binary variable regarding the conducting of the ceremony (1: municipality with the ceremony; 0: municipality without the ceremony). The impact of conducting the ceremony can be estimated with the coefficient $\gamma_{7}.$

### 2.4 Sub‐analysis and sensitivity analysis

In the sub‐analysis, we stratified each municipality into three groups by population size. We conducted DID and triple‐difference analyses to determine whether the impacts of the Coming‐of‐Age Day and ceremony differed by population size.

We conducted the analysis in two different ways for the sensitivity analysis. Since some municipalities conducted the Coming‐of‐Age ceremony on the day before the Coming‐of‐Age Day, we set the cumulative incidence of cases 4 days before and after the Coming‐of‐Age Day as the outcomes. Although the number of cases by date of onset is less affected by weekly bias than the number of cases by date of diagnosis, some weekend influence on the onset data may be present. To further reduce the effect of this bias, we set the seven‐day cumulative number of cases as the outcome instead of the four‐day cumulative number of cases.

### 2.5 Test for parallel trends

An essential assumption of DID estimation is that without treatment, the trend in outcomes would be the same for the treatment and control groups. Before the Coming‐of‐Age Day, if 20‐year‐olds had different epidemic dynamics than 19‐ and 21‐year‐olds, then the impact of the Coming‐of‐Age Day cannot be correctly estimated. We tested the parallel trends assumption by modifying the DID equation using only the data before the Coming‐of‐Age Day. Time was set as a binary variable for 1–4 days before or 5–8 days before the Coming‐of‐Age Day; the cumulative number of cases per population in each period was used as the outcome. By estimating the coefficients of the Time and Age interaction terms, we examined whether the pre‐Coming‐of‐Age Day trends were parallel. We considered *P* < 0.05 as a potentially significant interaction effect.

### 2.6 Ethics approval

The Ethics Review Board of the National Institute of Infectious Diseases reviewed this study; no ethical approval was necessary because this study was conducted for public health purposes using national surveillance data.

## 3 RESULTS

**FIGURE 1:**
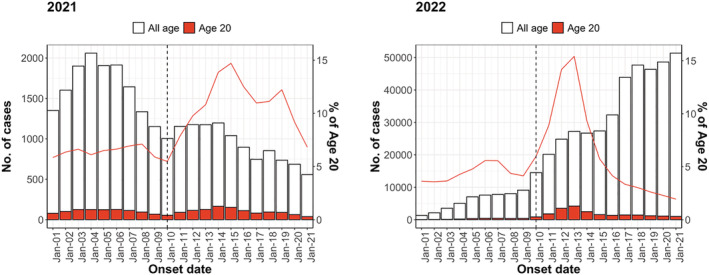
Epidemic curves for all age cohorts and 20‐year‐old age cohort in Japan in 2021 and January 2022. The red and black lines indicate the percentage of 20‐year‐old age cohort among all cases and the date of the Coming‐of‐Age Day, respectively.

**FIGURE 2:**
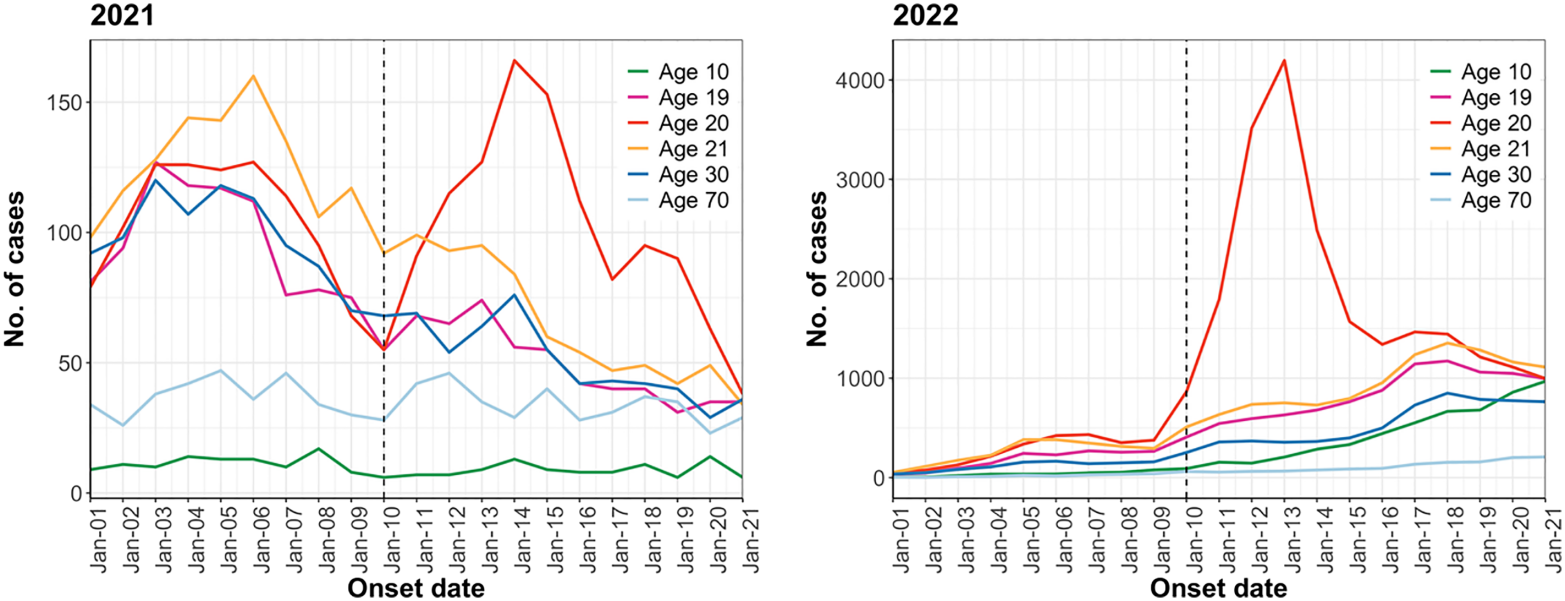
Epidemic curves for cases of the 10‐, 19‐, 20‐, 21‐, 30‐, and 70‐year‐old age cohorts in 2021 and 2022. The black line indicates the date of the Coming‐of‐Age Day.

We identified 35,264 and 26,474 onset cases in 2021 and 45,886 and 157,323 onset cases in 2022 in the week before (January 3 to January 9) and the week after (January 10 to January 16) the Coming‐of‐Age Day, respectively. Among 1916 municipalities, 73% (1397/1916) conducted the Coming‐of‐Age ceremony between January 8 and 10 in 2022. Although it was not possible to obtain the status of all municipalities in 2021, it was reported that many municipalities postponed their ceremonies due to the worsening of the COVID‐19 situation. There was a decreasing trend in 2021 and an increasing trend in 2022 in the number of reported cases. Both years showed an increase in the number and proportion of cases in the 20‐year‐old age cohort after the Coming‐of‐Age Day (Figure 1). The trend before the Coming‐of‐Age Day was almost the same for 19‐ and 21‐year‐olds as for the 20‐year‐old age cohort when compared with other age cohorts. Furthermore, relative to other age cohorts, the 20‐year‐old age cohort experienced a substantially larger increase after the Coming‐of‐Age Day, followed by a decline to the same level of reported cases in other age cohorts within approximately 7–10 days (Figure 2). The hypothesis that the pre‐exposure trend was statistically the same for the treatment and control groups was not rejected; the interaction term between Time and Age before the Coming‐of‐Age Day was not significant and was nearly 1 in both years (0.99 [95% confidence interval [CI] 0.80–1.22] in 2021 and 1.25 [95% CI 0.99–1.59] in 2022).

The primary analysis showed that the relative risks (RRs) of the Coming‐of‐Age Day were 1.27 (95% CI 1.02–1.57) in 2021 and 3.22 (95% CI 2.68–3.86) in 2022. Limiting to municipalities that conducted the Coming‐of‐Age ceremony, the RR of these municipalities was higher than that of municipalities that did not conduct the event (Table 1).

**TABLE 1:** Relative risk (RR) of the Coming‐of‐Age Day; for 2022, RRs are also shown stratified by whether or not the Coming‐of‐Age Ceremony was held

Year		RR
2022	All municipalities	3.22 (2.68–3.86)
	Municipalities with the ceremony	3.79 (3.15–4.57)
	Municipalities without the ceremony	1.34 (0.78–2.28)
2021	All	1.27 (1.02–1.57)

*Note*: Parentheses indicate 95% confidence intervals.

Table 2 shows the results of the sub‐analysis examining the impact of the Coming‐of‐Age Day when municipalities were stratified by population size. Even without considering the presence or absence of the Coming‐of‐Age ceremony in municipalities, the RR significantly increased among all groups in 2022. The magnitude of the impact of the Coming‐of‐Age Day was greater in municipalities with larger populations in 2022; however, we did not observe these trends in 2021. In 2022, when stratified by whether the Coming‐of‐Age ceremony was conducted, the impact of the Coming‐of‐Age Day was large (three‐fold increase or greater) and significant for all population groups in municipalities that conducted the ceremony. However, when limited to municipalities that did not conduct the ceremony, the RRs for the Coming‐of‐Age Day were markedly lower, with point estimates near 1 in municipalities with medium and smaller populations (RRs: 0.84 [95% CI 0.35–2.00] and 0.64 [95% CI 0.25–1.63] for medium‐ and small‐sized municipalities, respectively) (Table 2). In addition, the triple‐difference analysis showed that for all three population groups, the impacts of conducting the Coming‐of‐Age ceremony were large and significant, with RRs of 1.99–5.22 (Table 3).

**TABLE 2:** Relative risk (RR) of the Coming‐of‐Age Day by municipality size (municipalities categorized into three groups according to population size); for 2022, RRs are also shown stratified by whether or not the Coming‐of‐Age‐ceremony was held

Year	Municipality		RR
2022	Large municipalities	All	3.55 (2.85–4.44)
		With the ceremony	3.82 (3.06–4.77)
		Without the ceremony	1.92 (0.97–3.82)
	Medium municipalities	All	2.97 (2.17–4.07)
		With the ceremony	4.02 (2.87–5.61)
		Without the ceremony	0.84 (0.35–2.00)
	Small municipalities	All	1.98 (1.28–3.07)
		With the ceremony	3.35 (1.98–5.68)
		Without the ceremony	0.64 (0.25–1.63)
2021	Large municipalities	All	1.18 (0.94–1.49)
	Medium municipalities	All	1.50 (0.94–2.39)
	Small municipalities	All	2.35 (0.82–6.77)

*Note*: Parentheses indicate 95% confidence intervals.

**TABLE 3:** Relative risk (RR) of the Coming‐of‐Age‐ceremony for all municipalities and by municipality size (municipalities categorized into three groups according to population size) in 2022

Municipality	RR
All	2.80 (1.81–4.32)
Large municipalities	1.99 (1.11–3.55)
Medium municipalities	4.79 (2.32–9.91)
Small municipalities	5.22 (1.93–14.09)

*Note*: Parentheses indicate 95% confidence intervals.

Our sensitivity analysis showed similar trends for the aforementioned RRs. However, we observed two different results from the main analysis. First, when we set the day before the Coming‐of‐Age Day as the treatment day, the estimated RRs were smaller (e.g., RR of the Coming‐of‐Age Day in all municipalities in 2022 was 2.59 [95% CI 2.14–3.13]). Second, the RR for the seven‐day cumulative incidence was greater than that of the four‐day cumulative incidence in 2021, whereas the results were the opposite in 2022 (Tables S1–S3).

## 4 DISCUSSION

The present study showed the impact of the Coming‐of‐Age Day, a national holiday, on the population, specifically targeting only the 20‐year‐old age cohort in Japan. Our results indicated that the Coming‐of‐Age Day had a substantial impact on increasing viral transmission among the 20‐year‐old age cohort, regardless of the epidemic trend or population size, and that conducting the Coming‐of‐Age ceremony led to an additional risk of transmission. However, even when the ceremony was not conducted, the Coming‐of‐Age Day event itself may have contributed to the increase in the number of cases, as suggested by the following results: the point estimate of the RR for the Coming‐of‐Age Day restricted to municipalities without the ceremony in 2022 was 1.34 and not statistically significant, and the RR for the Coming‐of‐Age Day was significant in 2021, when many municipalities refrained from conducting the ceremony. Other data sources showing a sharp increase in university‐related clusters in the second and third weeks of 2022, followed by a sharp decline,^15^ also suggest the impact of the Coming‐of‐Age Day on these age groups.

The results of the sub‐analysis, stratified by population size, showed that the risk of the Coming‐of‐Age Day was greater in municipalities with smaller populations when the pandemic was declining (2021) and in municipalities with larger populations when the pandemic was increasing (2022). The results could be attributed to epidemic expansion beginning in densely populated areas of large cities.^16^

Notably, compared with the sensitivity analysis results, the RR of the cumulative number of cases in 4 days was greater than that of the cumulative number of cases in 7 days in 2022. In contrast, the converse was true in 2021. One possible explanation may be the difference in the SARS‐CoV‐2 variants prevalent in 2021 and 2022: the wild type was predominant in January 2021,^17^ whereas the Omicron type was predominant in 2022,^18^ with a shorter incubation period. This suggests that, in 2022, by 4 days after the Coming‐of‐Age Day, many cases developed symptoms due to exposure during the gathering. Furthermore, the RR was smaller when the day before the Coming‐of‐Age Day was set as the treatment date (January 9), suggesting that the majority of the impact of the Coming‐of‐Age Day was attributed to the number of cases with onset after January 10.

During the pandemic, many local governments reported conducting the Coming‐of‐Age ceremony after taking sufficient measures to prevent the spread of infection.^19^, ^20^, ^21^ Despite such efforts, the number of cases increased, especially in municipalities conducting the ceremony, suggesting that many infections did not spread during the ceremony but rather through small group gatherings and drinking parties after the ceremony. Similarly, in Scotland, where a sharp increase in infections was reported after international soccer matches, it was suggested that transmission occurred not at the official event venues, but as a result of more frequent social gatherings surrounding the matches.^4^ When conducting subsequent Coming‐of‐Age ceremonies, public health response could be improved by alerting individuals to undertake infection control measures during the ceremony and prevent the accompanying individual risk behaviors/activities following the ceremony.

We examined the impact of social gatherings on the epidemic expansion only in the 20‐year‐old age cohort; therefore, it is not possible to determine how the event affected the overall epidemic. Impressively, in Figure 2, the number of cases among the 20‐year‐old age cohort transiently surged after the Coming‐of‐Age Day but subsequently declined, finally reaching a level similar to that of the other age cohorts. Due to the transmission characteristics, where only a small percentage of cases cause secondary transmission,^22^, ^23^ the impact of this transient rise in the 20‐year‐old age cohort may not have such a large impact on the overall population. However, the transmission chain could continue as even larger clusters emerge from these infected individuals.^24^ This unique social natural experiment may have the potential to elucidate the dynamics of the transmission chain in more detail. Further studies are warranted on the chain of transmission after secondary cases on the Coming‐of‐Age Day.

Our study has several limitations. It is not possible in practice to confirm the violation of an important assumption in DID, the SUTVA (Stable unit treatment value assumption); observations in one unit should not be affected by a particular assignment of treatment to another unit. In our analysis, the 19‐ and 21 year‐old age cohorts, the control group, may have been affected by the Coming‐of‐Age Day. Although these populations are not the target of the celebrations, some individuals likely got infected from the 20‐year‐olds infected due to the Coming‐of‐Age Day. Moreover, some cases in the control group may have been exposed to risks related to the Coming‐of‐Age Day (e.g., having dinner with a 20‐year‐old sibling and serving a 20‐year‐old in a restaurant). Therefore, we referred to our results as impacts, not “causal effects.” Furthermore, to deal with the issue to the best extent possible, we set the outcome in the main analysis as the cumulative number of cases for a limited number of days (4 days). In addition, cases reported in a municipality that did not conduct the Coming‐of‐Age ceremony may have participated in the ceremony in their hometown; hence, the RR of the Coming‐of‐Age ceremony could have been underestimated. Moreover, our study employed the number of cases based on the onset date as the outcome. Although the analysis would have been more valid if the infection dates were known, we used the onset date due to the limitations in the surveillance data. There could be recall bias and social desirability bias when assessed based on disease onset data.^25^ Furthermore, there may have been differences in health‐seeking behavior between the treatment and control groups. If the treatment group, the new adults, had been more concerned and more likely to see a clinician or had been instructed to see a clinician with any concern, it is possible that the cases of new adults were more likely to be detected. This would lead to an overestimation of the RR of the Coming‐of‐Age Day and ceremony. Finally, since the results are based on the specific events made possible by the unique situation, it cannot be extrapolated to the risk of infection in other social gatherings. However, we provide empirical evidence for the simple but difficult‐to‐prove fact that gatherings increase the risk of infection.

In conclusion, we estimated the impact of the Coming‐of‐Age Day and ceremony on the number of incident infections. Given our results quantifying the increased risk of infections, discussions are needed on how the ceremony should be conducted during the pandemic and how infection can be avoided, rather than canceling the event. Furthermore, when planning large social events in the future, it is important to be aware of the unique risks associated with these gatherings and effectively communicate such information to the relevant persons and communities.

## CONFLICT OF INTEREST

The authors declare no competing interests.

## AUTHOR CONTRIBUTIONS

**Yura Ko:** Conceptualization; data curation; formal analysis; investigation; methodology; resources; visualization. **Ryo Kinoshita:** Conceptualization; investigation; methodology; validation. **Masato Yamauchi:** Data curation; investigation; resources. **Kanako Otani:** Data curation; investigation; resources. **Taro Kamigaki:** Project administration; resources; supervision; validation. **Kazuki Kasuya:** Investigation; resources. **Daisuke Yoneoka:** Formal analysis; investigation; methodology; supervision; validation. **Yuzo Arima:** Investigation; supervision; validation. **Yusuke Kobayashi:** Investigation; resources; validation. **Takeshi Arashiro:** Investigation; resources; validation. **Miyako Otsuka:** Investigation; resources; validation. **Reiko Shimbashi:** Investigation; resources; validation. **Motoi Suzuki:** Formal analysis; funding acquisition; investigation; project administration; resources; supervision; validation.

## Supporting information


**Supplementary Table 1.** Relative risk (RR) of the Coming‐of‐Age Day when A) setting the day before the Coming‐of‐Age Day as the treatment day and B) setting the cumulative number of cases as seven days instead of four days for the outcome. For 2022, RRs are also shown stratified by whether or not the Coming‐of‐Age ceremony was held. Parentheses indicate 95% confidence intervals.
Supplementary Table 2. Relative risk (RR) of the Coming‐of‐Age Day by municipality size (municipalities categorized into three groups according to population size) when A) setting the day before the Coming‐of‐Age Day as the treatment day and B) setting the cumulative number of cases as seven days instead of four days for the outcome. For 2022, RRs are also shown stratified by whether or not the Coming‐of‐Age ceremony was held. Parentheses indicate 95% confidence intervals.
Supplementary Table 3. Relative risk (RR) of the Coming‐of‐Age ceremony municipality size (municipalities categorized into three groups according to population size) in 2022 when A) setting the cumulative incidence before and after the day before the Coming‐of‐Age Day as outcomes and B) setting the cumulative number of cases as seven days instead of four days for the outcome. Parentheses indicate 95% confidence intervals.

## Data Availability

No additional data available.
