# Supplementary material for: Impact of the Coming‐of‐Age Day and ceremony on the risk of SARS‐CoV‐2 transmission in Japan: A natural‐experimental study based on national surveillance data
Source: Influenza Other Respir Viruses. 2022 Jul 27;16(6):1026–32. doi: 10.1111/irv.13027 (PMC9353391; doi:10.1111/irv.13027)
Supplement: Supplementary file 1 — Supplementary Table 1. Relative risk (RR) of the Coming‐of‐Age Day when A) setting the day before the Coming‐of‐Age Day as the treatment day and B) setting the cumulative number of cases as seven days instead of four days for the outcome. For 2022, RRs are also shown stratified by whether or not the Coming‐of‐Age ceremony was held. Parentheses indicate 95% confidence intervals. Supplementary Table 2. Relative risk (RR) of the Coming‐of‐Age Day by municipality size (municipalities categorized into three groups according to population size) when A) setting the day before the Coming‐of‐Age Day as the treatment day and B) setting the cumulative number of cases as seven days instead of four days for the outcome. For 2022, RRs are also shown stratified by whether or not the Coming‐of‐Age ceremony was held. Parentheses indicate 95% confidence intervals. Supplementary Table 3. Relative risk (RR) of the Coming‐of‐Age ceremony municipality size (municipalities categorized into three groups according to population size) in 2022 when A) setting the cumulative incidence before and after the day before the Coming‐of‐Age Day as outcomes and B) setting the cumulative number of cases as seven days instead of four days for the outcome. Parentheses indicate 95% confidence intervals. [file IRV-16-1026-s001.docx]

Table of Contents

[Supplementary Table 1 1](#_Toc105283931)

[Supplementary Table 2 2](#_Toc105283932)

[Supplementary Table 3 4](#_Toc105283933)

Supplementary Table 1

Relative risk (RR) of the Coming-of-Age Day when A) setting the day before the Coming-of-Age Day as the treatment day and B) setting the cumulative number of cases as seven days instead of four days for the outcome. For 2022, RRs are also shown stratified by whether or not the Coming-of-Age ceremony was held. Parentheses indicate 95% confidence intervals.

A)

| Year |  | RR |
| --- | --- | --- |
| 2022 | All municipalities | 2.59 (2.14–3.13) |
|  | Municipalities with the ceremony | 3.02 (2.50–3.67) |
|  | Municipalities without the ceremony | 1.46 (0.80–2.67) |
| 2021 | All municipalities | 0.98 (0.79–1.22) |

B)

| Year |  | RR |
| --- | --- | --- |
| 2022 | All municipalities | 2.87 (2.31–3.56) |
|  | Municipalities with the ceremony | 2.92 (2.48–3.44) |
|  | Municipalities without the ceremony | 1.44 (0.87–2.40) |
| 2021 | All municipalities | 1.73 (1.45–2.07) |

Supplementary Table 2

Relative risk (RR) of the Coming-of-Age Day by municipality size (municipalities categorized into three groups according to population size) when A) setting the day before the Coming-of-Age Day as the treatment day and B) setting the cumulative number of cases as seven days instead of four days for the outcome. For 2022, RRs are also shown stratified by whether or not the Coming-of-Age ceremony was held. Parentheses indicate 95% confidence intervals.

A)

| Year | Municipality | | RR |
| --- | --- | --- | --- |
| 2022 | Large municipalities | All | 2.76 (2.21–3.45) |
|  |  | With the ceremony | 2.91 (2.35–3.61) |
|  |  | Without the ceremony | 1.93 (0.93–4.02) |
|  | Medium municipalities | All | 2.61 (1.84–3.70) |
|  |  | With the ceremony | 3.59 (2.53–5.09) |
|  |  | Without the ceremony | 1.08 (0.38–3.06) |
|  | Small municipalities | All | 1.85 (1.18–2.92) |
|  |  | With the ceremony | 3.18 (1.81–5.59) |
|  |  | Without the ceremony | 0.87 (0.36–2.13) |
| 2021 | Large municipalities | All | 0.97 (0.77–1.21) |
|  | Medium municipalities | All | 1.01 (0.63–1.61) |
|  | Small municipalities | All | 1.19 (0.43–3.26) |

B)

| Year | Municipality | | RR |
| --- | --- | --- | --- |
| 2022 | Large municipalities | All | 2.84 (2.27–3.56) |
|  |  | With the ceremony | 2.95 (2.40–3.61) |
|  |  | Without the ceremony | 1.87 (0.92–3.80) |
|  | Medium municipalities | All | 2.47 (1.83–3.34) |
|  |  | With the ceremony | 3.15 (2.35–4.24) |
|  |  | Without the ceremony | 1.01 (0.36–2.78) |
|  | Small municipalities | All | 1.69 (1.18–2.42) |
|  |  | With the ceremony | 2.40 (1.55–3.73) |
|  |  | Without the ceremony | 0.86 (0.39–1.88) |
| 2021 | Large municipalities | All | 1.70 (1.39–2.08) |
|  | Medium municipalities | All | 1.93 (1.31–2.86) |
|  | Small municipalities | All | 1.51 (0.66–3.42) |

# Supplementary Table 3

Relative risk (RR) of the Coming-of-Age ceremony municipality size (municipalities categorized into three groups according to population size) in 2022 when A) setting the cumulative incidence before and after the day before the Coming-of-Age Day as outcomes and B) setting the cumulative number of cases as seven days instead of four days for the outcome. Parentheses indicate 95% confidence intervals.

A)

| Municipality | RR |
| --- | --- |
| Large municipalities | 1.51 (0.87–2.61) |
| Medium municipalities | 3.31 (1.57–7.00) |
| Small municipalities | 3.64 (1.35–9.79) |

B)

| Municipality | RR |
| --- | --- |
| Large municipalities | 1.57 (0.93–2.67) |
| Medium municipalities | 3.14 (1.61–6.12) |
| Small municipalities | 2.80 (1.23–6.38) |
